# Supplementary material for: Changes in reproductive behavior associated with the perception and individual experiences of the COVID-19 pandemic
Source: PLoS One. 2023 Jul 19;18(7):e0288833. doi: 10.1371/journal.pone.0288833 (PMC10355434; doi:10.1371/journal.pone.0288833)
Supplement: Attachment — Submitted filename: Response to Reviewers.docx [file pone.0288833.s001.docx]

I very much appreciate the comments from both reviewers who revealed a weakness of the manuscript, where the theoretical part dropped the reflection on the division of labor in the household focusing on childcare, which was already reflected in the introduction and conclusion of the manuscript.

Specifically:

**1)**

R1: *The findings and the discussion did not quite address this particular assumption of the authors though: "that social distancing has a positive effect on the fertility intentions of women and suggest that the corresponding increased need for caring for the child and the household affect primarily women, thus pressurizing women into reverting to the traditional family model." A better explanation for this assumption would be rather crucial for the paper, I believe.*

R2: *In the introduction, the study mentions that the pandemic restrictions "allowed for a deepening of relationships between partners and increased involvement of both parents in caregiving". However, there were many reports on increased household and caregiving burdens on women, and increased gender based violence in homes during the pandemic across high income and low income countries. These important factors which would impact reproductive choices and behaviours, while touched upon in the introduction, are not discussed in detail in the study.*

I reflected on these two points by rewriting the relevant paragraph in the theoretical framing of the study.

Original version: *However, the anti-pandemic measures limited these options to a significant extent, and it is not unreasonable to assume that for part of the population, starting (or expanding) a family moved up many people’s list of priorities. This assumption is supported by a study by Voicu and Badoi [13], which examines the possible effects of the pandemic on fertility from the viewpoint of gender roles. The authors assume that social distancing has a positive effect on the fertility intentions of women and suggest that the corresponding increased need for caring for the child and the household affect primarily women, thus pressurizing women into reverting to the traditional family model. On the other hand, limited opportunities for self-realization as well as changes in the life situations of individuals in terms both of the quality of the partnership [14,15] and employment and income stability [4] may result in not only in a preference for parenthood but also in the case of the deterioration may have acted to reduce or delay reproduction.*

New version: *However, the anti-pandemic measures limited these options to a significant extent, and it is not unreasonable to assume that for part of the population, starting (or expanding) a family moved up many people’s list of priorities. In addition to the change in priorities [13], the enhancement of the reconciliation of work and family responsibilities may have contributed positively to fertility attitudes, for example via the option to work from home [13,14] and the forced reduction of working hours, which comprised an official supportive measure in many European countries during the pandemic [13,15]. Voicu and Badoi [16] suggest that this reconciliation may have increased the fertility of women who were forced to stay at home to care for their children due to the pandemic, and thus were forced to take on the position of caregiver due to the reduced availability of formal and informal childcare services [5]. It should be noted here that although the extra demands for childcare impacted both mothers and fathers, most of the extra responsibilities were shouldered by women [17,18,19,20]. On the other hand, the extra burden of caring for children, together with the limited opportunities for self-realization, as well as changes in the life situations of individuals in terms both of the quality of the relationship [21,22] and employment and income stability [4] may have resulted not only in a preference for parenthood but also, in the case of the deterioration of conditions, may have acted to reduce or delay reproduction.*

I hope that now the consideration of the impact of household arrangements on fertility intentions is clear.

I have also paid close attention to the comment on domestic violence by the second reviewer; in studying the available literature, I am unable to determine for certain whether domestic violence affects partners' reproductive intentions. I am aware that there has been an increase in domestic violence during the pandemic (in the case of the Czech Republic through NGOs providing support), I believe this is part of the assessment of relationship quality, which is mentioned by the manuscript.

**2)**

R2: *The study mentions that the pandemic period saw a reduction in opportunity costs concerning career progression for women depending on occupation. Is the data collected in the study segregated by occupation type. Would be beneficial to substantiate this with study data.*

I completely understand this idea and it was one of my initial considerations as the GGS study has ISCO codes for individual respondents. In the final version of the manuscript, I moved away from assessing these individual characteristics (such as education or job position), partly because this would have reduced the sample so much that the test results would no longer be reliable, and secondly, it would still be burdened with considerable error, since the very determination of which occupations were advantaged or disadvantaged by the pandemic would be very difficult.

**3)**

R2: *Are there any long-term repercussions of the findings presented in the study? Or implications for policy?*

I would like to thank the reviewer for reminding me this. The following paragraph has been added to the Conclusion.

New paragraphs: *The covid-19 pandemic period significantly changed not only society as a whole but also the lives of individuals. The results presented revealed that the reproductive behavior of individuals may change during difficult times. Although the quality of interpersonal relationships affects the decision-making process, material insecurity also contributes significantly to the final decision. Thus, it is important that even in times of crisis, be it due to pandemics or adverse economic conditions, the availability of assistance measures for economically active individuals and families is not reduced.*
